# Research trends on anti-PD-1/PD-L1 immunotherapy for esophageal cancer: A bibliometric analysis

**DOI:** 10.3389/fonc.2022.983892

**Published:** 2022-11-17

**Authors:** Yuanyuan Yang, Feng Wang

**Affiliations:** Department of Oncology, The First Affiliated Hospital of Zhengzhou University, Zhengzhou, China

**Keywords:** bibliometrics, anti-PD-1/PD-L1, immunotherapy, esophageal cancer, CiteSpace, HistCite, VOSviewer, Web of Science (WOS)

## Abstract

**Objectives:**

The study aims to summarize publication characteristics of anti-programmed cell death protein 1 (PD-1)/programmed cell death 1 ligand 1 (PD-L1) immunotherapy for esophageal cancer and create scientific maps to explore hotspots and emerging trends with bibliometric methods.

**Methods:**

The publications between 2012 and 2021 were retrieved from the Web of Science Core Collection (WoSCC) on June 20, 2022. Bibliometric tools including HistCite, VOSviewer, and CiteSpace were used for statistical analysis. Data on the trend of the annual output, countries/regions, institutions, journals, authors, subject categories, keywords, and co-cited references were presented in this study.

**Results:**

A total of 552 publications written by 3,623 authors of 872 institutions, 44 countries/regions in 250 journals were included in the bibliometric study. China, USA and Japan were the key countries in this field. Kato Ken, Bang Yung-Jue, *Frontiers in Oncology*, *Journal of Clinical Oncology* and Natl Canc Ctr were the top 1 productive author, co-cited author, productive journal, co-cited journal and prolific institution, respectively. The top 4 most present keywords were esophageal cancer, immunotherapy, esophageal squamous cell carcinoma and PD-L1. Neoadjuvant chemotherapy, response, PD-1 blockade and CD8^+^ T cell were four latest research frontiers. The keywords reflected the progress from PD-1/PD-L1 expression to the clinical application of PD-1/PD-L1 inhibitors. The current researches mainly focus on neoadjuvant immunotherapy for esophageal cancer and development of biomarkers. Further research is warranted to determine effective predictive biomarkers or models, illustrate the molecular mechanism of combined treatment, and construct the optimal therapeutic strategy.

**Conclusions:**

This study visually analyzed the global trend and hotspots of anti-PD-1/PD-L1 immunotherapy for esophageal cancer over the past decade. The results could guide scientists to comprehensively understand the global frontiers and determine future directions.

## Introduction

Esophageal cancer is the eighth most common cancer and the sixth major cause of cancer-related death worldwide (1). In 2020, the world witnessed about 604,100 new cases and 544,100 deaths, equaling to the age-standardized morbidity and mortality rates of 6.3/100,000 and 5.6/100,000, respectively (2). Advanced esophageal squamous cell carcinoma (ESCC) is one of devastating tumors with the 5-year survival rate lower than 5% (3, 4). The etiology of esophageal cancer is not completely clear. Recognized risk factors include genetic predisposition, gastroesophageal reflux disease, alcohol consumption, smoking and obesity (5). The alternative clinical treatment for esophageal cancer mainly depends on the stage of the tumor and the specific condition of patients. For esophageal cancer, multidisciplinary approach is an effective strategy for managing this disease, which involves the use of surgery, radiotherapy, chemotherapy, targeted therapy, immunotherapy and other treatments (6). In recent years, the emergence of immunotherapy has brought new hope for esophageal cancer. With the recognition of tumor immunotherapy, the application of immune checkpoint inhibitors (ICIs) has gradually shifted from the back-line and second-line treatments to first-line and even perioperative treatments. PD-1/PD-L1 inhibitors have been approved to be used for the first-line treatment of advanced esophageal cancer, which significantly improves patient prognosis (7). The response rate of ICI alone in esophageal cancer varied from 9.9 to 33.3% in the reported studies (8).

A large number of articles regarding anti-PD-1/PD-L1 immunotherapy for esophageal cancer have been published in the past decade. However, no systematic analysis of the data in the available articles has been performed. The increasing number of publications makes it more necessary to illustrate the state of the development by bibliometric methods (9). Bibliometric analysis consists of the quantification and visualization of the data by applying mathematics and statistics (10). In this way, the research trend of the area can be objectively shown. The bibliometric analysis provides researchers valuable information on the development of a specific field from a macro perspective. The most important data source is the Web of Science Core Collection (WoSCC) database (11).

Up to now, there is no published bibliometric study has systematically evaluated the anti-PD-1/PD-L1 immunotherapy for esophageal cancer from 2012 to 2021. In this work, the research tendency and hotspots of anti-PD-1/PD-L1 immunotherapy for esophageal cancer were visually analyzed using HistCite, VOSviewer, and CiteSpace. The aim was to identify the characteristics of publications, build collaboration networks, present hot words, reveal research frontiers and direct the follow-up work (12, 13).

## Materials and methods

### Data source and search strategy

**Figure 1:**
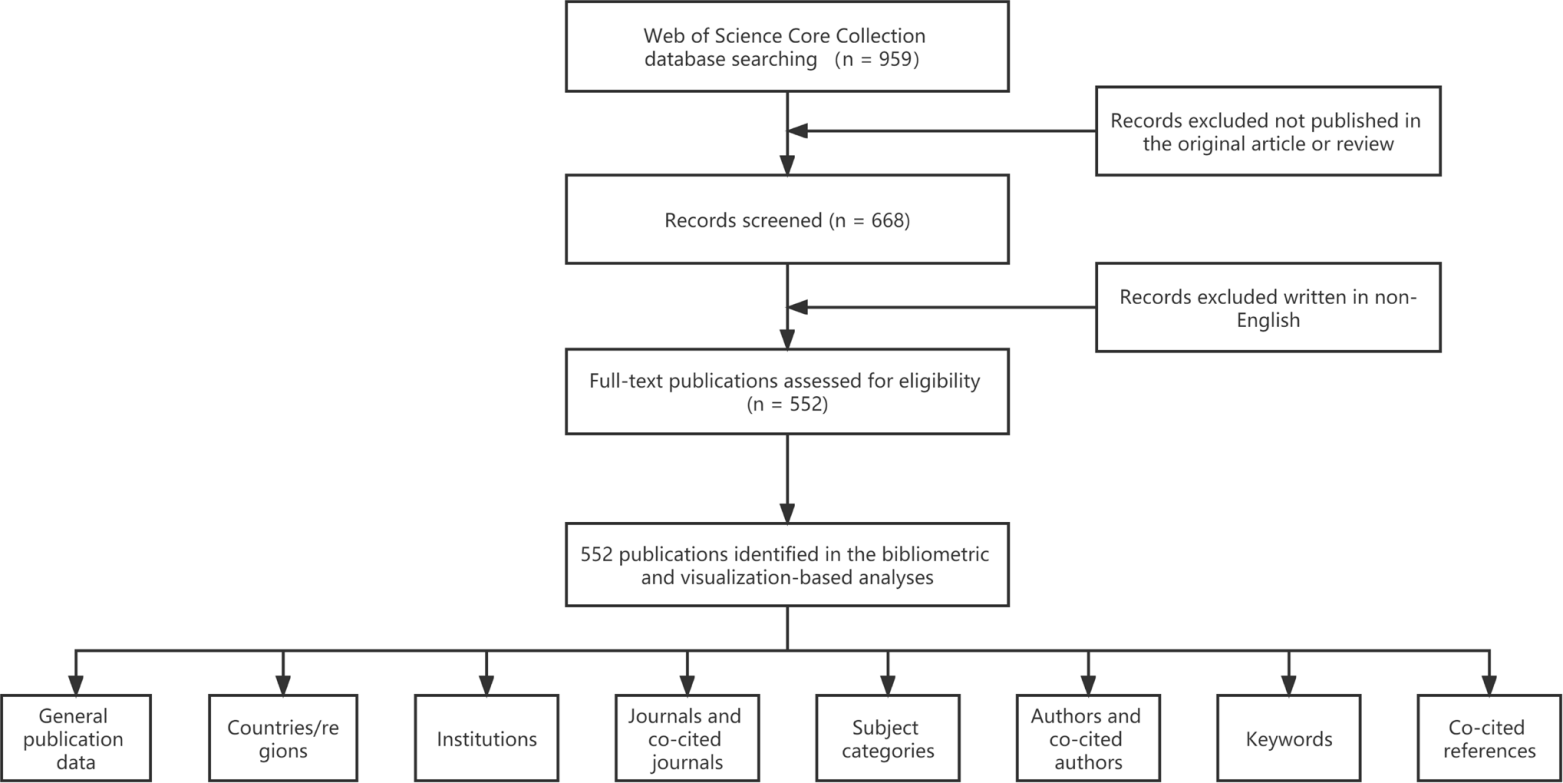
Flow Diagram of detailed retrieval process and analysis procedure.

The literature retrieval was performed online using the WoSCC database, which is the most influential citation academic document database worldwide, in order to collect publications on anti-PD-1/PD-L1 immunotherapy for esophageal cancer (14). The search was performed on June 20, 2022 to ensure the same conditions and avoid the bias resulting from daily updates (15). Since all data were downloaded from the public database, no ethical approval was needed for this work. The following formula was used to perform the advanced search: TS = ((esophageal neoplasm OR esophagus neoplasm OR esophageal cancer OR esophagus cancer OR esophageal tumor OR esophagus tumor OR esophageal carcinoma OR esophagus carcinoma OR ESCC) AND (programmed cell death 1 receptor OR programmed cell death ligand 1 OR CD274 OR B7H1 OR PD-1 OR PD-L1 OR Nivolumab OR Pembrolizumab OR Lambrolizumab OR Avelumab OR Atezolizumab OR Nivolizumab OR Durvalumab OR Pidilizumab OR Cemiplimab OR Camrelizumab OR Sintilimab OR Tisleizumab OR Toripalimab)). The detailed retrieval process and analysis procedure are shown in **Figure 1**.

### Inclusion criteria and exclusion criteria

The inclusion criteria are as follows: (a) literature on anti-PD-1/PD-L1 immunotherapy for esophageal cancer; (b) literature types include articles and reviews; (c) literature published between 2012 and 2021; (d) literature indexed in WoSCC.

The exclusion criteria are as follows: (a) Unpublished documents; (b) Duplicate reports.

### Statistical analysis

WoSCC was used to collect publications for bibliometric analysis and visualization. All the data retrieved from WoSCC were exported in plain text format. HistCite (Clarivate Analytics, Philadelphia, PA, the USA), VOSviewer 1.6.14 (Leiden University, Leiden, the Netherlands) and CiteSpace 5.3.R4 (Drexel University, Philadelphia, the USA) were used for statistical analysis (16). HistCite is a citation analysis software that summarizes and processes data quickly (17). In this study, annual output, language type, document type and total number of citations were analyzed by HistCite. VOSviewer is available for building and viewing bibliometric maps, and displays the results of the cluster analysis, including research characteristics, distribution and hotspots (18). The visual maps of countries/regions, institutions, journals, authors, keywords and references were generated by VOSviewer. CiteSpace, a Java application software, was used to explore the collaboration among countries/institutions/authors, identify co-cited authors/references, detect burst keywords and construct visualization maps (19). The software was effective in revealing the trends and dynamics of publications as well as capturing hotspots in a given research field (20). Due to its rich functions, CiteSpace has been widely used for bibliometric analysis. The CiteSpace parameters were as follows: time slicing (2012–2021), years per slice (1), term source (all selection), term type (burst terms), node type (choose one at a time), links (strength: cosine; scope: within slices), selection criteria (top 50 objects), and pruning (pathfinder and pruning sliced networks).

## Results

### General data and annual output

**Figure 2:**
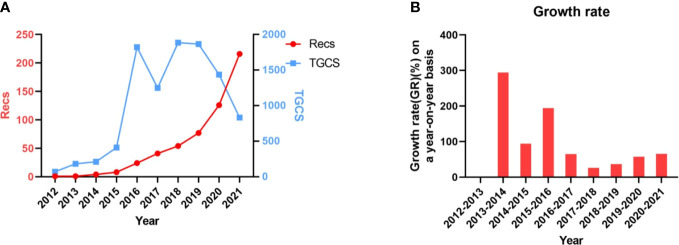
**(A**, **B)** The annual output, citations and growth rate presented by year from 2012 to 2021.

A total of 552 publications were included in the bibliometric study from 2012 to 2021. These publications were written by 3,623 authors of 872 institutions, 44 countries/regions in 250 journals, with 16,778 citations totally. All publications involved were made up of original articles (n = 410, 74.28%) and reviews (n = 142, 25.72%). The annual output generally maintained an increase trend in the past decade (**Figure 2**). The most prolific year was 2021 with 216 publications, while the minimum annual output occurred in 2012 with one article. 2014 is the fastest growing year in the past decade. As regard the total citations, the figure peaked at 1,886 in 2018 and bottomed out in 2012.

### Countries/regions

**Figure 3:**
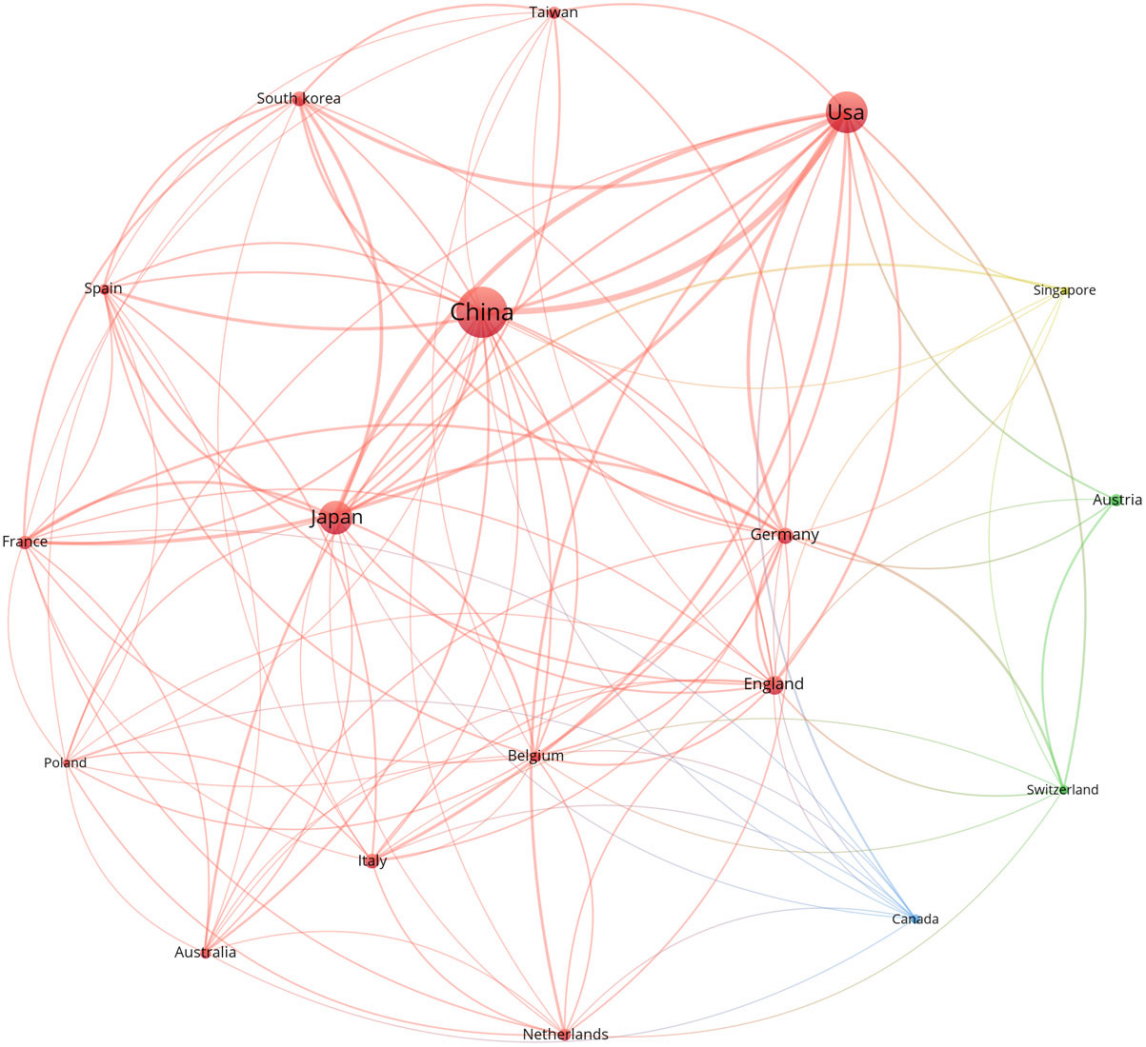
The co-authorship network visualization map of countries/regions. Larger nodes represent more publications of the term. Lines between nodes represent the connection between them.

A total of 44 countries/regions participated in the publication of anti-PD-1/PD-L1 immunotherapy for esophageal cancer in the last 10 years. Among them, China (n = 227, 32.76%) was the most prolific country/region, followed by the USA (n = 153, 22.08%) and Japan (n = 101, 14.57%). In terms of citations, the USA had the most total citations and France had the highest ratio of Citations/Paper. **Table 1** lists the top 8 most prolific countries/regions. A network map was constructed for countries with more than 5 publications. **Figure 3** shows that the map had 18 nodes. The 3 largest nodes respectively represented China, the USA and Japan for their huge number of publications. The USA had the most active cooperation with others, with the strongest total link strength (TLS, TLS = 125). The closest cooperation was between China and the USA (TLS = 27).

**Table 1:** The top 8 countries according to total publications from 2012 to 2022.

Rank	Country	Number of Publications	Proportion (%)	Total Citations	Citations/Paper
1	China	227	32.76%	3583	15.78
2	USA	153	22.08%	4562	29.82
3	Japan	101	14.57%	2406	23.82
4	England	30	4.33%	1147	38.23
5	Germany	25	3.61%	1145	45.80
6	South Korea	20	2.89%	738	36.90
7	Italy	20	2.89%	480	24.00
8	France	17	2.45%	920	54.12

Data were retrieved from 552 publications with VOSviewer on June 20, 2022.

### Institutions

**Figure 4:**
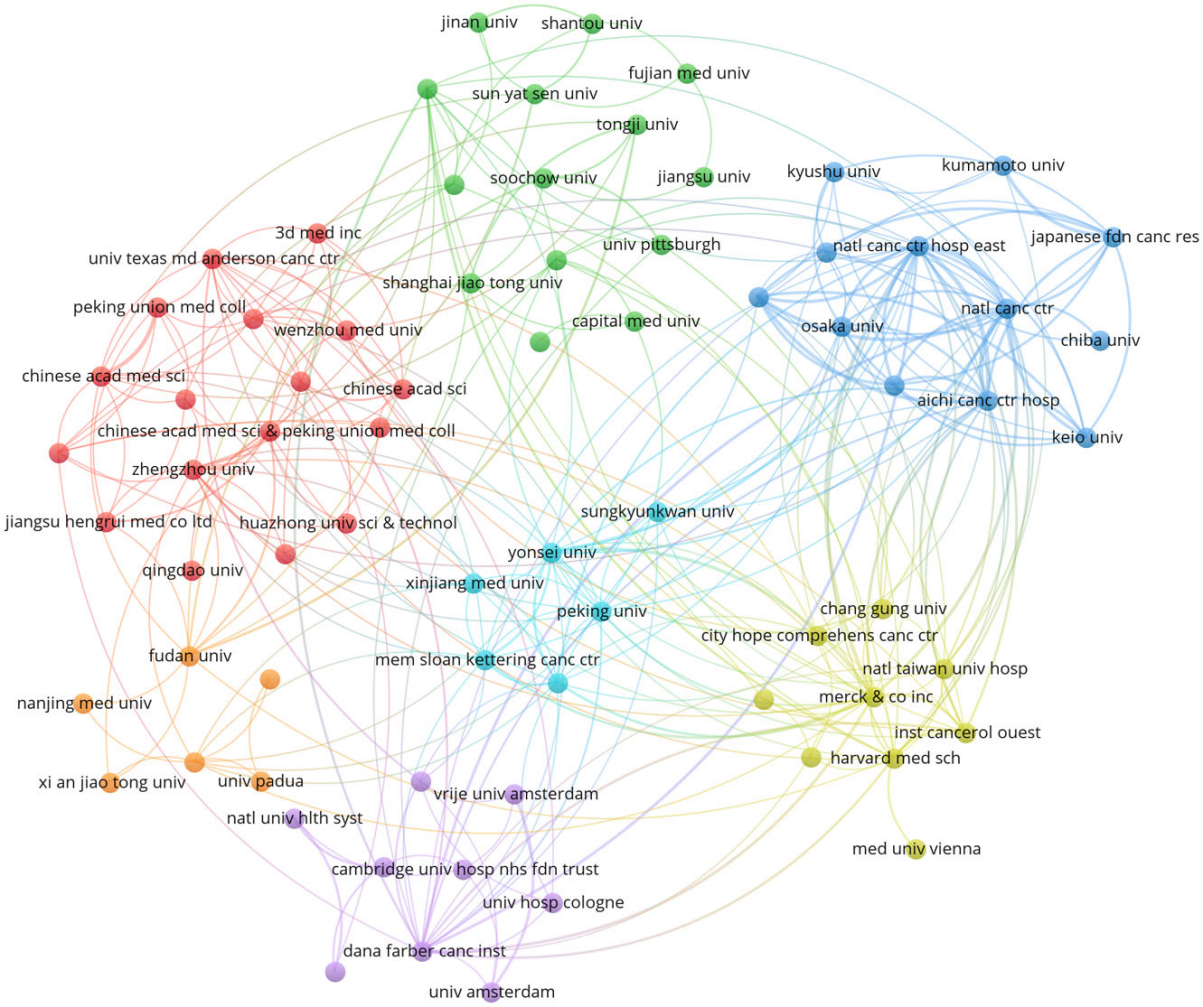
The co-authorship network visualization map of institutions.

A total of 872 institutions contributed to the research of anti-PD-1/PD-L1 immunotherapy for esophageal cancer. The top 10 most productive institutions from 2012 to 2021 are listed in **Table 2**. Natl Canc Ctr (Japan, 23 publications) and Zhengzhou Univ (China, 23 publications) tied first place, followed by Sun Yat Sen Univ (China, 21 publications), Natl Canc Ctr Hosp East (Japan, 19 publications) and Fudan Univ (China, 16 publications). The publications of the top 10 institutions accounted for more than 29% of the total publications. As regard the citations, Dana Farber Canc Inst (the USA, 1,108 citations) ranked first. Natl Canc Ctr Hosp East (Japan, 733 citations) and Natl Canc Ctr (Japan, 725 citations) came second and third, respectively. **Figure 4** shows the co-authorship network among institutions with 10 or more publications. It is evident that institutions in the same district always closely cooperate with each other. Natl Canc Ctr Hosp East (TLS = 78) had the most active cooperation with others. The closest cooperation was between Natl Canc Ctr Hosp East and Natl Canc Ctr (TLS = 14).

**Table 2:** The top 10 most productive institutions from 2012 to 2021.

Rank	The name of institution	Publications	Citations	Location
1	Natl Canc Ctr	23	725	Japan
2	Zhengzhou Univ	23	283	China
3	Sun Yat Sen Univ	21	168	China
4	Natl Canc Ctr Hosp East	19	733	Japan
5	Fudan Univ	16	85	China
6	Mem Sloan Kettering Canc Ctr	15	488	the USA
7	Soochow Univ	13	362	China
8	Dana Farber Canc Inst	12	1108	the USA
9	Chinese Acad Med Sci	12	284	China
10	Chinese Acad Med Sci & Peking Union Med Coll	12	58	China

Data were retrieved from 552 publications with VOSviewer on June 20, 2022.

### Journals and co-cited journals

All the 552 papers were published in 250 journals. The top 10 prolific journals and co-cited journals are listed in **Table 3**. The 10 most prolific journals published 164 papers, accounting for 29.71% papers involved in this study. The IF of these journals ranged from 3.111 to 13.801, half of which were higher than 6. Among these journals, *Frontiers in Oncology* (IF = 5.738; 22 publications) had most publications, followed by *Cancer Science* (IF = 6.518; 14 publications) and *Journal for Immunotherapy of Cancer* (IF = 12.469; 13 publications). The top 3 co-cited journals were as follows: *Journal of Clinical Oncology* (IF = 50.717; 1,788 co-citations), *New England Journal of Medicine* (IF = 176.079; 1,232 co-citations) and *Lancet Oncology* (IF = 54.433; 996 co-citations). The co-citations of the 50% of the listed journals were greater than 700 and 7 of the top 10 co-cited journals had IF higher than 50.

**Table 3:** The top 10 journals and co-cited journals on anti-PD-1/PD-L1 immunotherapy for esophageal cancer from 2012 to 2021.

Rank	Journal	Publication number	Citation	IF#	Co-cited journal	Co-citation	IF
1	Frontiers in Oncology	22	165	5.738	Journal of Clinical Oncology	1788	50.717
2	Cancer Science	14	434	6.518	New England Journal Of Medicine	1232	176.079
3	Journal for Immunotherapy of Cancer	13	298	12.469	Lancet Oncology	996	54.433
4	Annals of Translational Medicine	11	54	3.616	Clinical Cancer Research	853	13.801
5	Cancers	11	37	6.575	Annals of Oncology	787	51.769
6	Future Oncology	11	207	3.674	Lancet	591	202.731
7	Cancer Immunology Immunotherapy	10	142	6.630	Oncotarget	553	——
8	Clinical Cancer Research	8	812	13.801	Nature	552	69.504
9	Oncotargets and Therapy	8	456	4.345	Cancer Research	502	13.312
10	Oncology Letters	8	80	3.111	Science	352	63.714

Data were retrieved from 552 publications with VOSviewer on June 20, 2022.

#: Abbreviation for Impact Factor.

### Authors and co-cited authors

A total of 3,623 authors contributed to the involved publications. **Table 4** shows that the top 10 authors were all from Japan. Among them, Kato Ken with 16 publications and 410 citations was the most productive author, followed by Kojima Takashi (12 publications, 440 citations) and Doi Toshihiko (10 publications, 394 citations). As regard the co-cited authors, Bang Yung-Jue from South Korea ranked first with 197 co-citations, followed by Kato Ken (193 co-citations) and Fuchs Charles S. (184 co-citations).

**Table 4:** The top 10 prolific authors and co-cited authors on anti-PD-1/PD-L1 immunotherapy for esophageal cancer research from 2012 to 2021.

	Author				Co-cited authors		
Rank	Name	Publications	Citations	Country	Name	Co-citations	Country
1	Kato Ken	16	410	Japan	Bang Yung-Jue	197	South Korea
2	Kojima Takashi	12	440	Japan	Kato Ken	193	Japan
3	Doi Toshihiko	10	394	Japan	Fuchs Charles S.	184	the USA
4	Baba Hideo	8	222	Japan	Shah Manish A.	163	the USA
5	Yoshida Naoya	8	222	Japan	Le Dung T.	158	the USA
6	Doki Yuichiro	8	114	Japan	Janjigian Yelena Y.	154	the USA
7	Kono Koji	7	264	Japan	Shitara Kohei	134	Japan
8	Ishimoto Takatsugu	7	221	Japan	Kojima Takashi	125	Japan
9	Iwatsuki Masaaki	7	221	Japan	Kang Y.K.	117	South Korea
10	Mori Masaki	7	194	Japan	Topalian Suzanne L.	117	the USA

Data were retrieved from 552 publications with VOSviewer on June 20, 2022.

**Figure 5:**
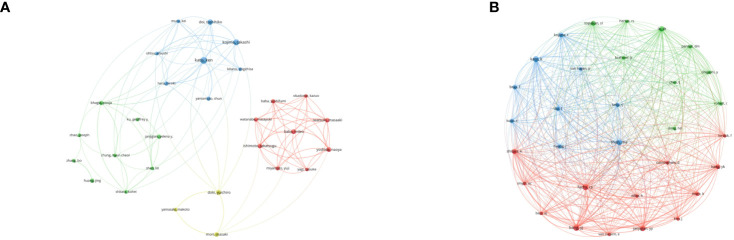
**(A)** Co-authorship network visualization map of authors. **(B)** Co-citation network visualization map of authors.

VOSviewer analyzed the information of authors and co-cited authors, then visualized it in a network map to explore influential researchers and potential collaborators (**Figure 5**) (21). The 42 authors with more than 5 publications formed several clusters and almost no collaboration was present among the clusters. The linkages among the authors were clearly less robust. However, the linkages among authors from same cluster were relatively close. When it comes to the co-cited authors, the minimum number of co-citations was set as 60. Unlike authors, the collaborations among 31 co-cited authors were quite active.

### Subject categories

**Figure 6:**
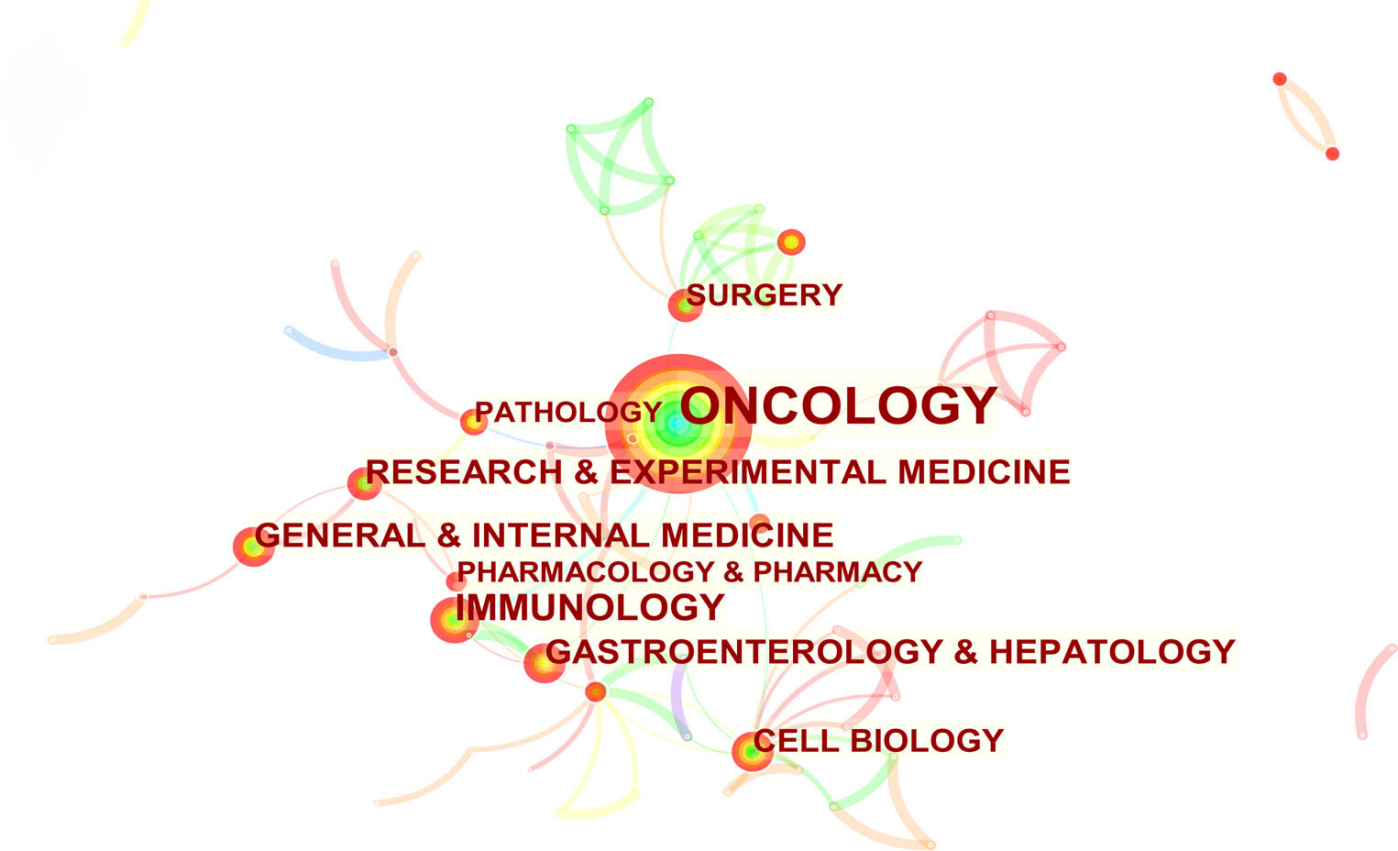
The visualization map of subject categories. The tree ring-shaped nodes represented different subject categories. The lines between two nodes meant co-occurrence. The area of the nodes referred to the number of publications. Nodes with high centrality were deemed as the hot field.

In the present study, CiteSpace was used to analyze the information regarding publication categories and construct a knowledge map (**Figure 6**). The larger node represented more publications of the term. Nodes with high centrality were usually considered as pivotal points in the field (22). In this work, the top 5 subject categories were selected according to the publication number and centrality. **Table 5** shows that ONCOLOGY and IMMUNOLOGY ranked first and second, respectively.

**Table 5:** Top 5 subject categories in terms of publication number and centrality related to anti-PD-1/PD-L1 immunotherapy for esophageal cancer.

Rank	Publications	Category	Centrality	Category
1	539	ONCOLOGY	1.32	ONCOLOGY
2	94	IMMUNOLOGY	0.82	RESEARCH & EXPERIMENTAL MEDICINE
3	74	RESEARCH & EXPERIMENTAL MEDICINE	0.72	CELL BIOLOGY
4	74	GENERAL & INTERNAL MEDICINE	0.66	PATHOLOGY
5	71	GASTROENTEROLOGY & HEPATOLOGY	0.42	SURGERY

Data were retrieved from 552 publications with CiteSpaceV on June 20, 2022.

### Keywords

**Figure 7:**
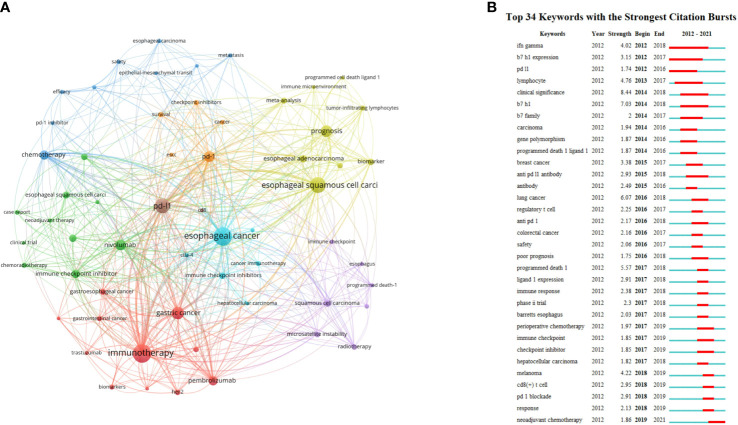
**(A)** The co-occurrence network visualization map of keywords. Keywords in the same color represent were sorted into the same cluster. **(B)** The top 34 keywords with the strongest citation bursts from 2012 to 2021. The red segment of the blue line denoted the burst duration of a keyword.

High-frequency keywords represent the hot topics in a particular field. Fifty-nine keywords with more than 5 occurrences were extracted from 552 publications. The top 4 keywords with most occurrences were listed as follows: esophageal cancer (n = 123), immunotherapy (n = 119), esophageal squamous cell carcinoma (n = 89), and PD-L1 (n = 79). VOSviewer was used to construct the network map of keywords, including esophageal cancer, immunotherapy, esophageal squamous cell carcinoma, PD-L1, PD-1, prognosis and so on (**Figure 7A**).

CiteSpace was used to identify and analyze keywords with citation bursts, thereby indicating the research hotspots and emerging trends over a period time. The minimum burst duration was set as 1 year. The top 34 keywords with the strongest citation burst are listed in **Figure 7B**. Among them, clinical significance had the highest burst strength (8.44). Response, PD-1 blockade, CD8^+^ T cell, and melanoma were the four keywords with the highest burst strength from 2018 to 2019. Neoadjuvant chemotherapy (NCT) was the top 1 keyword with the strongest citation bursts recently.

### Co-cited references

**Figure 8:**
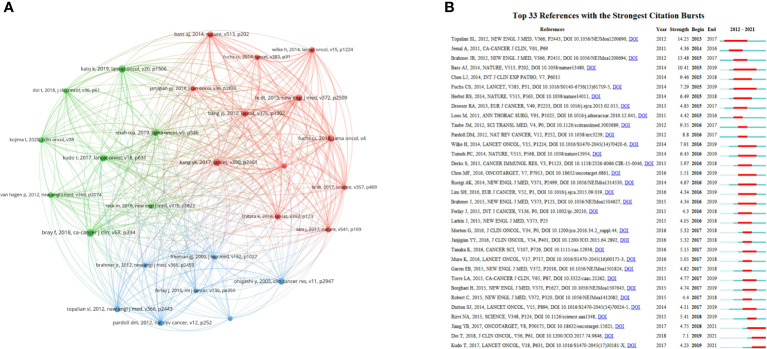
**(A)** The co-citation network visualization map of references from 2012 to 2021. **(B)** The top 33 references with the strongest citation bursts from 2012 to 2021. The red segment of the blue line denoted the burst duration of a keyword.

Co-cited reference is regarded as one of the most valuable indicators in bibliometrics that displays the key landmark articles of this field (23–25). **Table 6** lists the top 10 co-cited references. Among them, 8 articles were clinical trials, 2 were original articles. The article written by Freddie Bray et al. published in *CA Cancer J Clin* ranked first (n = 111) (26), followed by a clinical trial written by Yoon-Koo Kang et al. in *Lancet* (n = 99) (27) and another clinical trial written by Ken Kato et al. in *Lancet Oncol* (n = 97) (28). A co-citation network map was created using articles with more than 40 co-citations and explored the connection among these articles. The map contained 27 nodes, which clearly indicated the scientific relevance among these references. **Figure 8A** shows that the largest node represented the most co-cited reference. “Yoon-Koo Kang, 2017, *Lancet*, V390, P2461” (TLS = 606) (27) had the most active association with other references, followed by “Charles S Fuchs, 2018, *JAMA Oncol*, V4” (TLS = 529) (32) and “Manish A Shah, 2019, *JAMA Oncol*, V5, P546” (TLS = 480) (30).

**Table 6:** The top 10 co-cited reference from 2012 to 2021.

Rank	Co-cited reference	Count
1	Freddie Bray, 2018, CA Cancer J Clin, V68, P394 (26)	111
2	Yoon-Koo Kang, 2017, Lancet, V390, P2461 (27)	99
3	Ken Kato, 2019, Lancet Oncol, V20, P1506 (28)	97
4	Toshihiro Kudo, 2017, Lancet Oncol, V18, P631 (29)	91
5	Manish A Shah, 2019, JAMA Oncol, V5, P546 (30)	83
6	Dung T Le, 2015, New Engl J Med, V372, P2509 (31)	81
7	Charles S Fuchs, 2018, JAMA Oncol, V4 (32)	79
8	Yuichiro Ohigashi, 2005, Clin Cancer Res, V11, P2947 (33)	76
9	Yung Jue Bang, 2010, Lancet, V376, P1302 (34)	74
10	Suzanne L Topalian, 2012, New Engl J Med, V366, P2443 (35)	73

References with citation bursts refer to those that are frequently cited during certain a period of time (36). CiteSpace was used to perform references with citation bursts, and the minimum burst duration was set as 1 year. The blue line in **Figure 8B** represents the timeline in years, while the red line represents the time range in which a reference had citation burst (37). The burst strength of the top 33 references ranged from 4.05 to 14.25. Among them, “Topalian SL, 2012, *New Engl J Med*, V366, P2443 (35)“ had the highest burst strength (14.25), which ranked tenth in the list of co-citations, indicating the great influence of this study. The article assessed the antitumor activity and safety of anti–PD-1 antibody in cancer, showing that the adverse-event profile does not appear to preclude its use. “Kudo T, 2017, Lancet Oncol, V18, P631” (29), “Doi T, 2018, *J Clin Oncol*, V36, P61” (38) and “Jiang YB, 2017, *Oncotarget*, V8, P30175” (39) were 3 co-cited references with recent bursts. Toshihiro Kudo et al. conducted a phase II clinical trial and suggested that nivolumab exhibited favorable activity and controllable safety in ESCC (29). Toshihiko Doi et al. reported the results of KEYNOTE-028, a phase Ib study on PD-L1(+) patients with advanced solid tumors (38). Pembrolizumab displayed controllable toxicity and persistent antitumor activity in these patients. Yubo Jiang et al. revealed the prognostic significance of tumor-infiltrating immune cells and PD-L1 expression in ESCC (39).

## Discussion

Esophageal cancer has a high degree of malignancy and poor prognosis. As a new therapeutic method, immunotherapy can significantly improve the prognosis of patients (40, 41). The anti-PD-1/PD-L1 antibody is the most commonly used ICI. Therefore, it is important to build an in-depth understanding of publications in this field. In this study, a bibliometric analysis of anti-PD-1/PD-L1 immunotherapy for esophageal cancer from 2012 to 2021 was performed, presenting the research hotspots and trends.

**Figure 2** shows that the annual output maintained a rapid growth over the last decade. Literature published between 2012 and 2016 mostly focused on the expression and prognostic value of PD-1/PD-L1. In 2017, results of clinical trials of ICIs for esophageal cancer began to be published. From then on, the annual output increased rapidly, from 40 in 2017 to 216 in 2021. The annual growth rate also increased year by year.

In 2017, Toshihiko Doi (38) and Toshihiro Kudo (29) released their respective clinical trial results, which respectively demonstrated that Pembrolizumab and Nivolumab had certain anti-tumor effect in PD-L1(+) patients who failed second-line or back-like treatment. In 2018, Huang Jing et al. conducted a study on 30 patients with relapsed or metastatic advanced ESCC that showed chemoresistance previously (42). According to their results, the anti-PD-1drug SHR-1210 exhibited definite antitumor activity, with tolerable toxic and side effects. In 2019, the research data from multiple clinical trials were released, including KEYNOTE-180 (30), KEYNOTE-181 (43) and ATTRACTION-03 (28). As revealed by KEYNOTE-180 and KEYNOTE-181, Pembrolizumab had remarkable therapeutic effect and favorable safety on patients with PD-L1(+) advanced esophageal carcinoma, supporting the application of Pembrolizumab as the new second-line standard treatment for PD-L1(+) metastatic esophageal carcinoma. In 2019, Pembrolizumab was approved by the USA FDA to be used to treat relapsed, locally advanced or metastatic ESCC patients who had received first-line or later-line systemic treatment, with positive PD-L1 expression in tumor tissues (CPS≥10). In 2020, a breakthrough was made in the immunotherapy for esophageal carcinoma. The preliminary research results from KEYNOTE-590 demonstrated the satisfactory therapeutic effect and safety of Pembrolizumab combined with chemotherapy in the first-line treatment for advanced esophageal carcinoma (44). In the 2020 V5 version of NCCN guidelines, Pembrolizumab combined with platinum-based chemotherapeutic regimens was recommended in the first-line treatment of unresectable, locally advanced, locally relapsed or metastatic esophageal carcinoma with PD-L1 CPS≥10 and negative HER-2 expression. Additionally, CheckMate-577 ushered in the new chapter of adjuvant immunotherapy for esophageal carcinoma, which comprehensively evaluated the therapeutic effect of adjuvant nivolumab on patients with esophageal carcinoma and gastroesophageal junction carcinoma who did not achieve complete pathological remission after neoadjuvant radiochemotherapy (NRCT) (45). Clinical trials of neoadjuvant immunotherapy, such as NICE, KEEP-G 03, and PALACE-1, also reported the preliminary results. In March 2021, based on the KEYNOTE-590 research results, the USA FDA approved the use of Pembrolizumab combined with platinum-based chemotherapy as the first-line treatment for unresectable locally advanced or metastatic esophageal carcinoma or gastrointestinal junction carcinoma or those not suitable for radical radiochemotherapy, regardless of the PD-L1 expression status. The results of neoadjuvant immunotherapy combined with chemotherapy or radiochemotherapy were also released in 2021. The rapid development of immunotherapy for esophageal cancer suggests the great potential of the field in the future. Given that the feasibility and safety of anti-PD-1/PD-L1 immunotherapy have been confirmed, there might be more publications in the following years. The development prospects of immunotherapy for esophageal cancer could be expected.

China was the top 1 country ranked by total publications, which was consistent with epidemiological status that the incidence rate and fatality rate were high in this country. Although China had a huge number of publications, its citation was not impressive. Germany and France with less publications had high ratio of Citations/Paper, reflecting the high quality of their publications. The USA was the most active collaborator in **Figure 3** and played an important role in international cooperation.

As China is one of the high-risk areas of esophageal cancer, 6 of the top 10 institutions are from China. However, the articles published in China were scarcely cited, reflecting the weak influence of these publications. Therefore, Chinese institutions need to find methods to improve the quality of publications.

Natl Canc Ctr and Natl Canc Ctr Hosp East located in Japan were institutions that not only productive but also influential. They were both participating institutions of several important clinical trials, including KEYNOTE-180, KEYNOTE-181 and KEYNOTE-590. Ken Kato from National Cancer Center Hospital together with Toshihiko Doi and Takashi Kojima from National Cancer Center Hospital East were all contributed to the three clinical trials. They were also the top 3 prolific authors. Kato Ken who was the most prolific author ranked second in terms of co-cited authors, and he was a key figure of ATTRACTION-3. The top 10 prolific authors were all from Japan, indicating that Japanese scientists made a tremendous contribution in this field.

The analysis of prolific journals guides scientists in identifying core journals for information access and manuscript submissions. Three journals were highly recommended to scientists in the field: *Frontiers in Oncology*, *Cancer Science* and *Journal for Immunotherapy of Cancer*. Moreover, *Journal of Clinical Oncology*, *Lancet Oncology*, and *New England Journal of Medicine*, were the most authoritative journals in this field according to the co-citation amount shown in **Table 3**. As regard the subject categories shown in **Figure 6** and **Table 5**, ONCOLOGY and IMMUNOLOGY occupied central positions in this field, which were consistent with the analyzing results of the journals.

The current research hotspots were obtained from the high frequency keywords and cited references, which helped researchers to rapidly understand the direction of the research. This work lists the remarkable highlights of the research field as follows.

Clinical significance had the highest burst strength among the 34 keywords. It has always been significant in the field from 2014 to 2018. During this period of time, many studies focused on the prognostic value of PD-1/PD-L1 in patients with esophageal cancer. Numerous studies have suggested that, PD-L1 expression is related to the adverse clinical outcomes of esophageal cancer, supporting its role as a prognostic biomarker (39, 46–49). Further study found that PD-L1 expression in ESCC tumor cells was significantly associated with worse survival while no statistical significance was found between PD-L1 expression in ESCC tumor-infiltrating immune cells and survival (50). Recently, Peipei Wang et al. claimed that increased co-expression of PD-L1 and TIM3/TIGIT was associated with poor overall survival of ESCC (51).

Neoadjuvant chemotherapy was the hottest keywords in the last two years. With the moving forward of immunotherapy, more and more publications about NCT or NRCT plus immunotherapy are available at present. Multiple studies have evaluated the safety, feasibility and efficacy of neoadjuvant PD-1/PD-L1 inhibitors combined with chemotherapy in treating esophageal cancer patients (52–62). The neoadjuvant treatment of PD-1/PD-L1 inhibitor with chemotherapy produced satisfactory outcomes, indicating its potential as a promising neoadjuvant treatment for esophageal cancer. Besides, Wenqun Xing et al. designed a study to explore the impact of chemotherapy and toripalimab sequence on the pathological complete response (pCR) rate and safety of locally advanced ESCC patients (63). The initial results showed that delaying toripalimab to day 3 in chemoimmunotherapy might achieve a higher pCR rate than that on the same day. In the PERFECT trial, we investigated the feasibility and efficacy of NCRT combined with PD-L1 inhibitor (64). However, most of these studies were single-arm, phase I or II clinical trials. The long-term efficiency of this novel treatment and the validity of the present findings should be confirmed with more large-scale, longer follow-up and prospective comparative trials. In the existing clinical trials on neoadjuvant immunotherapy for esophageal carcinoma, no definite molecular biomarkers are available for selecting the possibly beneficial population. The previous research mainly focused on PD-L1, TMB, EGFR and CD8+ T cells. These studies not only help to identify the molecular biomarkers, but also provide ideas for the design of phase III clinical trials. Further studies should confirm more predictive biomarkers as well as indicators for the selection of a specific treatment.

The application of PD-1/PD-L1 inhibitors in esophageal cancer has achieved unprecedented successes. However, some treated patients exhibit non-response and severe immune-related adverse events. Therefore, immunotherapeutic markers are needed to assist in screening populations who can gain benefits from immunotherapy. At present, PD-L1 expression is used as the major biomarker for efficacy prediction in the application of PD-L1 inhibitors (65). With the deepening of research, DNA mismatch repair-deficient/microsatellite instability (dMMR/MSI) (66), tumor mutational burden (TMB) (67), copy number variation (CNV), polymerase epsilon (POLE) (68, 69), circulating tumor DNA (ctDNA) (70), inflamed gene expression profile (71, 72), tumor-infiltrating lymphocytes (TILs) (73), and immune gene signatures (74) have been suggested to show certain potential in predicting efficacy, which deserve further verification. Park R et al. Mentioned that, for esophageal cancer, there is no highly sensitive or specific marker apart from dMMR/MSI-H (75). Consequently, it is necessary to develop biomarkers for immunotherapy.

For the time being, the sensitivity and specificity of single biomarkers are not high enough. As a result, they may not be used as biomarkers alone. The combination of multiple biomarkers contributes more to predicting the immunotherapeutic efficacy in esophageal cancer. Moreover, when immunotherapy is used in combination with other treatments, whether a specific biomarker can maintain its prediction ability should be further analyzed. Whether predictors verified in advanced tumors can be applied in perioperative treatment is another important problem to be answered by on-going and future trials. In the future, it is promising to develop more precise tools to predict the anti-PD-1/PD-L1 therapeutic efficacy in esophageal cancer patients by standardizing and normalizing diverse biomarkers, intensively investigating the relations of different biomarkers, and applying computer technologies and medical databases, which is of great significance for individualized immunotherapy.

As far as we know, this is the first bibliometric study regarding anti-PD-1/PD-L1 immunotherapy for esophageal cancer in the past decade. The article hopes to guide scholars select research direction, references, cooperative institutions and authoritative journals. The data analysis was relatively objective and comprehensive, clearly displaying the research status visually. However, here are some limitations as follows.

1. The study included articles and reviews retrieved from WoSCC. Articles of other types or from other databases could not be involved in our study, thus limit the comprehensiveness of the study.
2. Papers published from 2012 to 2021 were retrieved on June 20, 2022. However, the database is still updating the data. Therefore, some recent publications could not be included. Besides, the number of citations of recent literature might be affected.
3. All of the publications included were in English, which might lead to a linguistic bias. Languages like Chinese, Japanese, French, German, Polish, Hungarian, Portuguese, Rumanian and Korean were not involved in the database. Therefore, it is likely that our results may not be applicable to publications in other languages.
4. Although analysis process was performed by software objectively, the method to explain these results had inherent subjective bias by individuals.

Still, it is believed that this article provides the overall situation and research trend of anti-PD-1/PD-L1 immunotherapy for esophageal cancer. The results could provide readers a general overview of the landscape, especially to those without in-depth knowledge. The information could also be used to explore possible collaboration partners, potentially relevant publications, and promising research directions. Our study not only exhibits important milestones of esophageal cancer immunotherapy but also offers a better guide to the future. We sincerely hope that bibliometric and visual analyses will give us more ideas in this field.

## Conclusion

To conclude, this article provides a comprehensive understanding of publications on anti-PD-1/PD-L1 immunotherapy for esophageal cancer from 2012 to 2021, providing valuable information to researchers in this field. This article presents data on the trend of annual output, countries/regions, institutions, journals, authors, subject categories, keywords, and co-cited references obtained using bibliometric analysis. Neoadjuvant chemotherapy, response, PD-1 blockade and CD8+ T cell were four latest research frontiers. Further studies and more cooperation are needed worldwide. Overall, our results could help the discovery of new perspectives and determine future directions.

## Data availability statement

Data were retrieved from 552 publications with VOSviewer on June 20, 2022. The datasets presented in this study can be found in online repositories. The names of the repository/repositories and accession number(s) can be found in the article/ supplementary material.

## Author contributions

Study conception, design and data analysis: YY. Paper writing: YY. Language polishing, paper review and editing: FW. All authors read and approved the submitted version. All authors contributed to the article and approved the submitted version.

## Funding

This study was supported by Health Training Program Foundation for Young and Middle-Aged Innovative Talents of Science and Technology (YXKC2020017) and Programs for Medical Science and Technology Development of Henan Province of China (SBGJ202002080).

## Acknowledgments

We thank Danyang Chen for assistance with manuscript preparation.

## Conflict of interest

The authors declare that the research was conducted in the absence of any commercial or financial relationships that could be construed as a potential conflict of interest.

## Publisher’s note

All claims expressed in this article are solely those of the authors and do not necessarily represent those of their affiliated organizations, or those of the publisher, the editors and the reviewers. Any product that may be evaluated in this article, or claim that may be made by its manufacturer, is not guaranteed or endorsed by the publisher.
